# Long-term care utilization within older adults with schizophrenia: Associated factors in a multicenter study

**DOI:** 10.1192/j.eurpsy.2023.1980

**Published:** 2023-07-19

**Authors:** L. Pierre, M. Kibby, M. Sanchez Rico, C. Hanon, J. Alvarado, R. Pascal de Raykeer, F. Limosin, N. Hoertel

**Affiliations:** 1 Psychiatry, APHP, Paris, France; 2 Department of Psychiatry and Behavioral Sciences, Duke University Medical Center, Durham, United States; 3 Department of Psychiatry, AP-HP Center, Issy-les-Moulineaux, France; 4 Department of Psychobiology & Behavioral Sciences Methods, Universidad Complutense de Madrid, Madrid, Spain; 5 INSERM 1266, INSERM, Paris, France

## Abstract

**Introduction:**

Data are scarce regarding the clinical factors associated with utilization of long-term care facilities among older adults with schizophrenia.

**Objectives:**

In this multicenter study, we sought to examine potential clinical differences between older adults with schizophrenia who are living in a long-term care facility and their community-dwelling counterparts.

**Methods:**

We used data from the French Cohort of individuals with Schizophrenia Aged 55-years or more (CSA) study, a large multicenter sample of older adults with schizophrenia (N = 353).

We used data from the French Cohort of individuals with Schizophrenia Aged 55-years or more (CSA)study, a large multicenter sample of older adults with schizophrenia (N = 353).

**Results:**

Results from the multivariable binary logistic regression analysis including all variables that had a significant association in univariate analyses (i.e., p < 0.05) revealed that older age (Adjusted odds ratio (AOR) [95%CI]=1.08 [1.03–1.13]), depression (AOR [95%CI]=1.97 [1.06–3.64]), lower MMSE (AOR [95%CI]=0.94 [0.88–0.99]) and GAF scores (AOR [95%CI]=0.97 [0.95–0.99]), living in an area comprising more than 1000 inhabitants per km2 (AOR [95%CI]=2.81 [1.37–5.80]), having consulted a general practitioner in the past year (AOR [95%CI]=0.28 [0.0.14–0.56]), and a greater lifetime number of hospitalizations in a psychiatric department (AOR [95%CI]=2.30 [1.18–4.50]) were significantly and independently associated with long-term care utilization among older adults with schizophrenia . In the multivariable logistic regression model, the variance inflation factor (VIF) and tolerance values of each predictor variable were respectively lower than 2.5 and higher than 0.2, supporting that multicollinearity was not a concern in our analysis.

**Conclusions:**

In a multicenter sample of 353 older adults with schizophrenia, we found that ong-term care utilization was significantly and independently associated with depression, lower cognitive and global functioning, greater lifetime number of hospitalizations in a psychiatric department, not having consulted a general practitioner in the past year, urbanicity and older age. Patients living in a long-term care facility appear to belong to a distinct group, marked by a more severe course of illness with higher level of depression and more severe cognitive deficits.

Despite its limitations, this study contributes to gain more specific knowledge about this specific understudied population. Our study highlights the need of early assessment and management of depression and cognitive deficits in this population and the importance of monitoring closely this vulnerable population.

**Disclosure of Interest:**

None Declared

